# Gender differences in the mediation of BMI and hepatic steatosis by sex hormones: Insights from NHANES 2017 to 2023

**DOI:** 10.1097/MD.0000000000044864

**Published:** 2025-10-31

**Authors:** Mingxing Zhao, Caifeng Yang

**Affiliations:** a Department of Gastroenterology, Xi’an No.1 Hospital, The First Affiliated Hospital of Northwest University, Xi’an, Shaanxi Province, China; b Department of Gastroenterology, Xi’an International Medical Center Hospital, Xi’an, Shaanxi Province, China.

**Keywords:** BMI, gender differences, hepatic steatosis, mediation analysis, sex hormones

## Abstract

Obesity, often assessed by body mass index (BMI), is closely associated with hepatic steatosis, but the role of sex hormones in mediating this relationship, particularly between genders, is not fully understood. Using National Health and Nutrition Examination Survey data from 2017 to 2023, we investigated gender differences in how sex hormones mediate the relationship between BMI and hepatic steatosis. A total of 9383 participants were included, with controlled attenuation parameter (CAP) used to assess liver fat accumulation. Nine sex hormones were measured, and relevant covariates were adjusted for, employing weighted multivariate linear regression to identify the mediators between BMI and hepatic steatosis. BMI was positively correlated with hepatic steatosis in both men and women. Mediation analysis showed that sex hormones mediated the relationship between BMI and hepatic steatosis. In males, hormones such as 17α-hydroxyprogesterone (2.18%), estrone sulfate (0.88%), estrone (0.43%), follicle-stimulating hormone (FSH) (0.08%), and sex hormone-binding globulin (SHBG) (6.95%) played a role. In females, 17α-hydroxyprogesterone (0.25%), progesterone (0.39%), FSH (0.53%), luteinizing hormone (0.31%), and SHBG (8.15%) mediated the association. Our study reveals that sex hormones play a key role in mediating the relationship between BMI and hepatic steatosis in a gender-specific manner.

**Key Points**

1. Sex-specific mediation: Sex hormones mediate the BMI–hepatic steatosis link differently in males and females, with SHBG (6.95% mediation in males, 8.15% in females) as the strongest shared mediator.
2. Gender-divergent pathways: In males, estrone sulfate (0.88%) and estrone (0.43%) drive mediation; in females, progesterone (0.39%) and luteinizing hormone (0.31%) play unique roles.
3. Large-scale evidence: Analysis of 9383 NHANES participants (2017–2023) using transient elastography (CAP) for precise liver fat quantification.
4. Clinical relevance: Findings clarify why males are more prone to hepatic steatosis and highlight potential targets for gender-specific interventions.

## 1. Introduction

Obesity, commonly measured using body mass index (BMI) – the most widely employed metric – has emerged as a major public health challenge, affecting over 650 million adults worldwide according to World Health Organization estimates,^[1]^ and is closely linked to a range of metabolic disorders.^[2–4]^ One significant consequence of obesity is hepatic steatosis, also known as fatty liver disease, which is characterized by the abnormal accumulation of fat within liver cells. Hepatic steatosis is associated with an increased risk of progressive liver damage, as it can advance to conditions such as steatohepatitis, cirrhosis, and systemic metabolic dysfunction, and it is also linked to a higher likelihood of developing hepatocellular carcinoma.^[5,6]^ A growing body of evidence highlights the role of sex hormones as key regulators of metabolic processes, including lipid homeostasis,^[7,8]^ energy expenditure,^[9]^ and liver function.^[10]^ Sex hormones are primarily steroid-based, including androgens (e.g., testosterone), estrogens (e.g., estradiol), and progestogens (e.g., progesterone), while their regulation involves peptide gonadotropins (e.g., follicle-stimulating hormone (FSH), luteinizing hormone (LH)) and carrier proteins like sex hormone-binding globulin (SHBG), which modulate hormone bioavailability. Sex hormones display distinct gender-based differences: androgens are typically more abundant in males, estrogens predominate in premenopausal females. Sex hormones exert a pivotal role in adipose metabolism and lipid metabolism.^[11]^ Evidence indicates that BMI is linked to alterations in sex hormone levels,^[12,13]^ which subsequently contribute to the development of hepatic steatosis.^[14,15]^ In other words, sex hormones may mediate the relationship between BMI and liver fat accumulation; however, this relationship, including its gender-specific differences, remains not fully understood. To further investigate the relationship, we conducted a study using data from the National Health and Nutrition Examination Survey (NHANES) spanning from 2017 to 2023. The introduction of advanced assessment techniques, such as liver ultrasound transient elastography for measuring liver fat content, incorporated by NHANES in 2017, provides a solid foundation for our analysis, facilitating more precise evaluations. Our study aimed to investigate gender differences in how sex hormones mediate the relationship between BMI and hepatic steatosis, thereby deepening our understanding of the underlying pathways and potentially guiding targeted therapeutic strategies.

## 2. Methods

### 2.1. Data and characteristics

**Figure 1.**
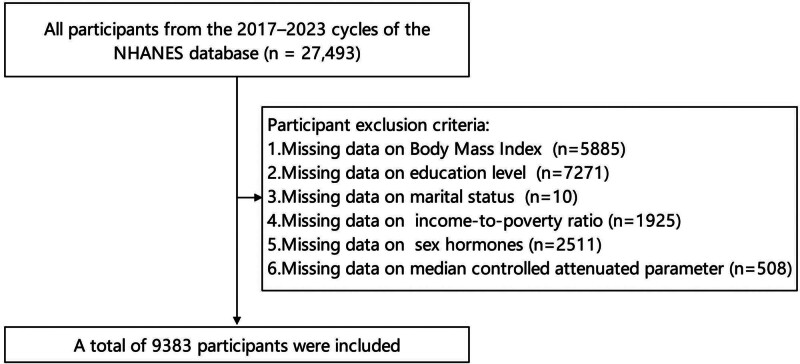
Flowchart of participant inclusion criteria and screening process of the 27,493 NHANES participants reviewed, 9383 were included in the final analysis after excluding those with missing data. NHANES = National Health and Nutrition Examination Survey.

The NHANES survey, which has been systematically collecting data on the health, nutrition, and socio-economic status of the U.S. population for over 4 decades, is a carefully constructed and ongoing research initiative. Its primary aim is to deliver comprehensive, objective insights into the nation’s health conditions while identifying emerging health issues. To achieve a representative sample of the U.S. population, NHANES employs a multi-stage, stratified, and clustered probability sampling method. A notable strength of this methodology is the targeted oversampling of specific groups, including older adults and racial/ethnic minorities, ensuring their adequate representation in the findings. During the 2017 to 2020 and 2021 to 2023 NHANES cycles, new assessment methods were introduced, including ultrasound technology and vibration-controlled transient elastography for measuring the controlled attenuated parameter (CAP) as an indicator of liver fat content, as well as expanded hormone measurements, such as SHBG, progesterone, estrone, and 17α-hydroxyprogesterone. In light of the introduction of new liver assessment methods and hormone measurements, this study was conducted to investigate whether BMI influences the risk of liver fat accumulation by affecting hormone levels, ultimately involving 9383 eligible participants, with the inclusion and exclusion criteria presented in Figure 1 providing a comprehensive outline of the participant selection process. This study was conducted in accordance with the Declaration of Helsinki. Data for this analysis were sourced from the NHANES database, which is publicly accessible and de-identified, eliminating the need for additional ethical approval or participant consent.

### 2.2. Variables

To assess hepatic steatosis and quantify liver fat content, the median CAP is employed, with elevated values signifying increased fat accumulation in the liver. To ensure the reliability of these measurements, NHANES enforces strict quality control protocols, including maintaining a ratio of interquartile range to median below 30%, based on a minimum of 10 measurements, thus ensuring precise and consistent results. To assess the role of hormones in liver fat content and their potential mediation of the relationship between BMI and hepatic steatosis, we included measurements of 9 sex hormones in this study: 17α-hydroxyprogesterone, androstenedione, estradiol, estrone, estrone sulfate, FSH, LH, progesterone, and SHBG. Additionally, our study focused on 2 main categories of factors: BMI as the independent variable and a set of covariates, including marital status, race, education level, age, gender, and the poverty-income ratio.

### 2.3. Statistical analysis

Given the complex multistage probability sampling design of the NHANES database, TST special subsample weights^[16]^ was incorporated into the analysis. Statistical significance was defined as a two-tailed *P* value of <.05. For continuous variables that were skewed or non-normally distributed, weighted medians and weighted interquartile ranges were used to summarize the characteristics of the data, while categorical variables were described using unweighted frequencies and weighted percentages; to account for the complex survey design, Pearson χ^2^ test with Rao & Scott adjustment was applied to categorical variables, and the design-based Kruskal–Wallis test was used to compare continuous variables across groups. To accurately represent the target population, we performed weighted multivariate linear regression analyses, employing techniques from the survey package^[17]^ that accounted for stratification, clustering, and sampling weights. Mediational relationships were assessed following the framework outlined by Baron and Kenny,^[18]^ where the independent variable is first shown to affect both the mediator and the dependent variable, the mediator influences the dependent variable, and the effect of the independent variable on the dependent variable is reduced upon controlling for the mediator, indicating the presence of a mediating effect. Statistical analyses were conducted exclusively using R software (version 4.3.2), which is available at http://www.R-project.org.

## 3. Results

### 3.1. Study population demographics

Following rigorous screening, a total of 9383 participants were retained for the study (see Table 1). Among these, 3416 individuals (weighted: 36.8%) were classified as having no steatosis (CAP < 238 dB/m), whereas 5967 participants (weighted: 63.2%) were identified as having steatosis (CAP ≥ 238 dB/m). It is important to note that the sample sizes in Table 1 are unweighted counts, whereas all reported proportions, means, standard deviations, medians, quartiles, and between-group differences were computed using weighted statistics to account for the sampling design. Overall, the gender distribution was approximately balanced, with 50.4% females and 49.6% males. However, the no steatosis group comprised a significantly higher proportion of females (57.3%), while the steatosis group included more males (53.6%), with the difference reaching statistical significance (*P* < .001). The overall mean age of the cohort was 47.8 years (standard deviations = 17.1). In subgroup analyses, the no steatosis group had a median age of 41 years (interquartile range [IQR]: 29–59), whereas the steatosis group had a significantly higher median age of 51 years (IQR: 36–63; *P* < .001). The racial composition of the cohort was predominantly non-Hispanic White (63.2%), followed by Hispanic (15.3%), non-Hispanic Black (10.7%), and non-Hispanic Asian and other (10.8%), with significant differences observed between groups (*P* < .001). Additionally, education levels and marital status differed significantly between groups (both *P* < .001), while the poverty income ratio did not vary significantly (*P* = .3).

**Table 1:** Characteristics of participants.

Characteristic	Overall, N = 9383 (100.0%)*	No steatosis (CAP < 238 dB/m), N = 3416 (36.8%)†	Steatosis (CAP ≥ 238 dB/m), N = 5967 (63.2%)†	*P*-value‡
Gender				
Female	4909 (50.4%)	1936 (57.3%)	2973 (46.4%)	<.001
Male	4474 (49.6%)	1480 (42.7%)	2994 (53.6%)	
Age (yr)	47.8 (17.1)	41.0 (29.0, 59.0)	51.0 (36.0, 63.0)	<.001
Race/Ethnicity				
Non-Hispanic White	4522 (63.2%)	1602 (62.6%)	2920 (63.6%)	<.001
Hispanic	1808 (15.3%)	555 (13.2%)	1253 (16.5%)	
Non-Hispanic Black	1693 (10.7%)	724 (13.0%)	969 (9.4%)	
Non-Hispanic Asian and other Race	1360 (10.8%)	535 (11.3%)	825 (10.5%)	
Education level				
9–11th grade	821 (6.2%)	294 (5.7%)	527 (6.4%)	<.001
College graduate or above	2965 (34.1%)	1264 (40.2%)	1701 (30.6%)	
High school graduate/GED	2054 (25.9%)	671 (22.9%)	1383 (27.6%)	
<9th grade	510 (3.1%)	150 (2.8%)	360 (3.3%)	
Some college or AA degree	3033 (30.7%)	1037 (28.4%)	1996 (32.1%)	
Marital status				
Married/living with partner	5439 (62.5%)	1817 (55.9%)	3622 (66.4%)	<.001
Never married	1807 (19.9%)	857 (27.0%)	950 (15.7%)	
Widowed/divorced/separated	2137 (17.6%)	742 (17.1%)	1395 (17.9%)	
Poverty income ratio	3.14 (1.63)	3.31 (1.67, 5.00)	3.17 (1.71, 5.00)	.3

The sample sizes for each group are unweighted, while the proportions, mean, standard deviation, median, quartiles, and differences between groups are all calculated using weighted statistics.

* n (unweighted) (%); Mean (SD).

† n (unweighted) (%); Median (Q1, Q3).

‡ Pearson χ^2^: Rao & Scott adjustment; design-based Kruskal–Wallis test.

### 3.2. BMI, sex hormones, and hepatic steatosis

We conducted a multivariable weighted analysis, accounting for the influence of covariates, to examine the relationship between BMI and hepatic steatosis across both males and females, as well as in each gender separately. As shown in Table S1 (Supplemental Digital Content, https://links.lww.com/MD/Q408), we found that BMI is a risk factor for hepatic steatosis (median CAP): in both males and females (beta = 5.19, *P* < .0001), in males (beta = 6.09, *P* < .0001), and in females (beta = 4.63, *P* < .0001).

**Figure 2.**
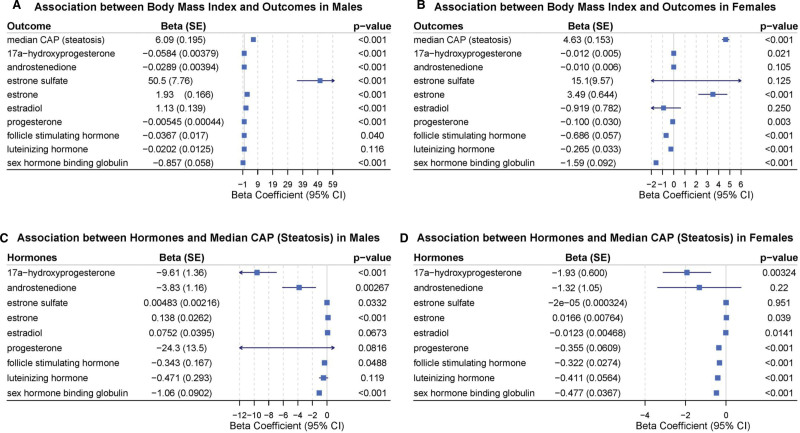
Forest plots illustrating associations between body mass index, sex hormones, and hepatic steatosis (A) and (B) present forest plots of the associations between body mass index (BMI) and outcomes (median controlled attenuation parameter [CAP, indicating steatosis] and 9 sex hormones) in males and females, respectively, with values of beta coefficient (standard error, SE) and corresponding *P*-values shown; (C) and (D) display forest plots of the associations between 9 sex hormones and median CAP (steatosis) in males and females, respectively, also including beta coefficient (SE) and *P*-value data. All associations are derived from weighted multivariate linear regression analyses adjusted for relevant covariates (marital status, race, education level, age, and poverty-income ratio), and the x-axis represents the beta coefficient with 95% confidence interval (95% CI). These results correspond to the data presented in Table 2 (associations between BMI and sex hormones/median CAP) and Table 3 (associations between sex hormones and median CAP) of the main text. CAP = controlled attenuated parameter.

As shown in Table S2 (Supplemental Digital Content, https://links.lww.com/MD/Q408), we conducted a further multivariable weighted linear regression analysis, accounting for the influence of covariates, to explore the relationship between BMI and 9 sex hormones. For clarity, the results of BMI and each hormone are presented separately in Table 2, with covariates not being included in this specific presentation. We found that in males, BMI was positively correlated with estrone sulfate (beta = 50.5, *P* < .0001), estrone (beta = 1.93, *P* < .0001), and estradiol (beta = 1.13, *P* < .0001), negatively correlated with 17α-hydroxyprogesterone (beta = −0.0584, *P* < .0001), androstenedione (beta = −0.0289, *P* < .0001), progesterone (beta = −0.00545, *P* < .0001), FSH (beta = −0.0367, *P* = .0402), and SHBG (beta = −0.857, *P* < .0001), and showed no association with LH (beta = −0.0202, *P* = .116). We found that in females, BMI was positively correlated with estrone (beta = 3.49, *P* < .0001) and negatively correlated with 17α-hydroxyprogesterone (beta = −0.0119, *P* = .0213), progesterone (beta = −0.100, *P* = .00265), FSH (beta = −0.686, *P* < .0001), LH (beta = −0.265, *P* < .0001), and SHBG (beta = −1.59, *P* < .0001), while no association was observed with androstenedione (beta = −0.00972, *P* = .105), estrone sulfate (beta = 15.1, *P* = .125), or estradiol (beta = −0.919, *P* = .250). The associations of BMI with median CAP (steatosis) and the 9 sex hormones in males and females (consistent with Table 2 data) are visually presented via forest plots in Figure 2A and B, respectively.

**Table 2:** Weighted multivariable linear regression analysis of BMI and covariates on median controlled attenuated parameter (steatosis) and 9 sex hormones in males and females.

Serial number	Males					Females				
	Dependent variable	Independent variable	Beta	SE	*P*-value	Dependent variable	Independent variable	Beta	SE	*P*-value
1	Median CAP (steatosis) in males	Body mass index	6.09E+00	1.95E−01	2.45E−23	Median CAP (steatosis) in females	Body mass index	4.63E+00	1.53E−01	6.28E−23
2	17α-Hydroxyprogesterone in males	Body mass index	−5.84E−02	3.79E−03	3.23E−15	17α-Hydroxyprogesterone in females	Body mass index	−1.19E−02	4.90E−03	2.13E−02
3	Androstenedione in males	Body mass index	−2.89E−02	3.94E−03	5.37E−08	Androstenedione in females	Body mass index	−9.72E−03	5.79E−03	1.05E−01
4	Estrone sulfate in males	Body mass index	5.05E+01	7.76E+00	4.66E−07	Estrone sulfate in females	Body mass index	1.51E+01	9.57E+00	1.25E−01
5	Estrone in males	Body mass index	1.93E+00	1.66E−01	3.04E−12	Estrone in females	Body mass index	3.49E+00	6.44E−01	9.07E−06
6	Estradiol in males	Body mass index	1.13E+00	1.39E−01	8.04E−09	Estradiol in females	Body mass index	−9.19E−01	7.82E−01	2.50E−01
7	Progesterone in males	Body mass index	−5.45E−03	4.38E−04	6.34E−13	Progesterone in females	Body mass index	−1.00E−01	3.04E−02	2.65E−03
8	Follicle stimulating hormone in males	Body mass index	−3.67E−02	1.70E−02	4.02E−02	Follicle stimulating hormone in females	Body mass index	−6.86E−01	5.70E−02	1.37E−12
9	Luteinizing hormone in males	Body mass index	−2.02E−02	1.25E−02	1.16E−01	Luteinizing hormone in females	Body mass index	−2.65E−01	3.30E−02	9.61E−09
10	Sex hormone binding globulin in males	Body mass index	−8.57E−01	5.76E−02	8.01E−15	Sex hormone binding globulin in females	Body mass index	−1.59E+00	9.18E−02	1.66E−16

Results of the weighted multivariable linear regression analysis of BMI and covariates on median CAP (steatosis) and 9 sex hormones in males (left) and females (right). Only the results for BMI in the multivariable analysis are shown; further details can be found in Table S2, Supplemental Digital Content, https://links.lww.com/MD/Q408.

CAP = controlled attenuated parameter.

**Table 3:** Weighted multivariable linear regression analysis of 9 sex hormones and covariates on median controlled attenuated parameter (steatosis) in males and females.

Serial number	Males					Females				
	Dependent variable	Independent variable	Beta	SE	*P*-value	Dependent variable	Independent variable	Beta	SE	*P*-value
1	Median CAP (steatosis) in males	17α-Hydroxyprogesterone	−9.61E+00	1.36E+00	1.06E−07	Median CAP (steatosis) in females	17α-Hydroxyprogesterone	−1.93E+00	6.00E−01	3.24E−03
2	Median CAP (steatosis) in males	Androstenedione	−3.83E+00	1.16E+00	2.67E−03	Median CAP (steatosis) in females	Androstenedione	−1.32E+00	1.05E+00	2.20E−01
3	Median CAP (steatosis) in males	Estrone sulfate	4.83E−03	2.16E−03	3.32E−02	Median CAP (steatosis) in females	Estrone sulfate	−2.00E−05	3.24E−04	9.51E−01
4	Median CAP (steatosis) in males	Estrone	1.38E−01	2.62E−02	1.35E−05	Median CAP (steatosis) in females	Estrone	1.66E−02	7.64E−03	3.90E−02
5	Median CAP (steatosis) in males	Estradiol	7.52E−02	3.95E−02	6.73E−02	Median CAP (steatosis) in females	Estradiol	−1.23E−02	4.68E−03	1.41E−02
6	Median CAP (steatosis) in males	Progesterone	−2.43E+01	1.35E+01	8.16E−02	Median CAP (steatosis) in females	Progesterone	−3.55E−01	6.09E−02	2.88E−06
7	Median CAP (steatosis) in males	Follicle stimulating hormone	−3.43E−01	1.67E−01	4.88E−02	Median CAP (steatosis) in females	Follicle stimulating hormone	−3.22E−01	2.74E−02	2.47E−12
8	Median CAP (steatosis) in males	Luteinizing hormone	−4.71E−01	2.93E−01	1.19E−01	Median CAP (steatosis) in females	Luteinizing hormone	−4.11E−01	5.64E−02	6.21E−08
9	Median CAP (steatosis) in males	Sex hormone binding globulin	−1.06E+00	9.02E−02	2.25E−12	Median CAP (steatosis) in females	Sex hormone binding globulin	−4.77E−01	3.67E−02	2.20E−13

Results of the weighted multivariable linear regression analysis of 9 sex hormones and covariates on median CAP (steatosis) in males (left) and females (right). Only the results for 9 sex hormones in the multivariable analysis are shown; further details can be found in Table S3 (Supplemental Digital Content, https://links.lww.com/MD/Q408).

CAP = controlled attenuated parameter.

As shown in Table S3 (Supplemental Digital Content, https://links.lww.com/MD/Q408), we conducted a multivariable weighted analysis, accounting for the influence of covariates, to examine the relationship between 9 sex hormones and median CAP in both males and females. For clarity, the results of the relationship between sex hormones and median CAP are presented separately in Table 3, with covariates excluded from this specific presentation. We found that in males, estrone sulfate (beta = 0.00483, *P* = .0332) and estrone (beta = 0.138, *P* < .0001) were positively correlated with median CAP, whereas 17α-hydroxyprogesterone (beta = −9.61, *P* < .0001), androstenedione (beta = −3.83, *P* = .00267), FSH (beta = −0.343, *P* = .0488), and SHBG (beta = −1.06, *P* < .0001) were negatively correlated with median CAP, and estradiol (beta = 0.0752, *P* = .0673), progesterone (beta = −24.3, *P* = .0816), and LH (beta = −0.471, *P* = .119) showed no association with median CAP. We found that in females, estrone (beta = 0.0166, *P* = .0390) is positively correlated with median CAP, while 17α-hydroxyprogesterone (beta = −1.93, *P* = .00324), estradiol (beta = −0.0123, *P* = .0141), progesterone (beta = −0.355, *P* < .0001), FSH (beta = −0.322, *P* < .0001), LH (beta = −0.411, *P* < .0001), and SHBG (beta = −0.477, *P* < .0001) are negatively correlated with median CAP, and no correlation was observed between androstenedione (beta = −1.32, *P* = .220), estrone sulfate (beta = −0.0000200, *P* = .951), and median CAP. The associations between each of the 9 sex hormones and median CAP (steatosis) in males and females (matching Table 3 results) are illustrated in Figure 2C and D.

### 3.3. Sex hormones as mediators of BMI and hepatic steatosis

We identified sex hormones that mediate the relationship between BMI and hepatic steatosis using 3 criteria: BMI is significantly correlated with these hormones; these hormones are significantly correlated with median CAP; and after controlling for these hormones, the relationship between BMI and median CAP should weaken, reflecting the influence of BMI on median CAP through these hormones. For example, if BMI is negatively correlated with SHBG and SHBG is negatively correlated with median CAP, adjusting for SHBG should reduce the positive correlation between BMI and median CAP. After meeting these criteria, we measured the mediating effect by calculating the percentage change in the beta value of BMI and median CAP after accounting for the influence of sex hormones, relative to the beta value when the hormones were not considered. We used multivariate weighted analysis to assess the impact of sex hormones on the relationship between BMI and median CAP, as shown in Table S4 (Supplemental Digital Content, https://links.lww.com/MD/Q408). To facilitate presentation, we extracted some of the results from the analysis – specifically, the effects of BMI and each sex hormone on median CAP in the multivariate analysis – into Table 4. We found that 6 out of the 9 hormones in males were significantly correlated with both BMI and median CAP. The beta value for the relationship between BMI and median CAP decreased after adjusting for 5 hormones, suggesting that these hormones mediate the relationship between BMI and hepatic steatosis. The hormones that mediate this effect in males include 17α-hydroxyprogesterone (mediating effect as a percentage of total effect: 2.18%), estrone sulfate (0.88%), estrone (0.433%), FSH (0.081%), and SHBG (6.95%). We also found that in females, 6 out of the 9 hormones included in our analysis were simultaneously correlated with both BMI and median CAP. Five of these hormones were found to mediate the relationship between BMI and median CAP, namely 17α-hydroxyprogesterone (0.252%), progesterone (0.394%), FSH (0.528%), LH (0.305%), and SHBG (8.15%). It is important to note that in males, BMI is negatively correlated with androstenedione, and androstenedione is also negatively correlated with median CAP; however, after adjusting for androstenedione, the positive correlation between BMI and median CAP became stronger. Similarly, estrone exhibited a similar pattern in females; therefore, these 2 hormones are not considered mediators of the relationship between BMI and median CAP.

**Table 4:** Weighted multivariable linear regression analysis of BMI, 9 sex hormones, and covariates on median controlled attenuated parameter (steatosis) in males and females.

Serial number	Males						Females					
	Dependent variable	Independent variable	Beta	SE	*P*-value	Ratio of BMI’s effect on median CAP (adjusted/unadjusted for sex hormones)	Dependent variable	Independent variable	Beta	SE	*P*-value	Ratio of BMI’s effect on median CAP (adjusted/unadjusted for sex hormones)
1	Median CAP (steatosis) in males	Body mass index	5.96E+00	1.99E−01	3.14E−22	97.824%	Median CAP (steatosis) in females	Body mass index	4.62E+00	1.55E−01	3.62E−22	99.748%
		17α-Hydroxyprogesterone	−2.27E+00	9.97E−01	3.09E−02	NA		17α-Hydroxyprogesterone	−9.77E−01	3.61E−01	1.17E−02	NA
2	Median CAP (steatosis) in males	Body mass index	6.10E+00	1.97E−01	1.17E−22	100.153%	Median CAP (steatosis) in females	Body mass index	4.63E+00	1.53E−01	2.42E−22	100.001%
		Androstenedione	3.22E−01	6.84E−01	6.42E−01	NA		Androstenedione	3.81E−03	5.02E−01	9.94E−01	NA
3	Median CAP (steatosis) in males	Body mass index	6.04E+00	1.99E−01	2.01E−22	99.120%	Median CAP (steatosis) in females	Body mass index	4.64E+00	1.52E−01	1.91E−22	100.107%
		Estrone sulfate	1.06E−03	1.48E−03	4.80E−01	NA		Estrone sulfate	−3.29E−04	2.38E−04	1.78E−01	NA
4	Median CAP (steatosis) in males	Body mass index	6.07E+00	1.88E−01	4.16E−23	99.567%	Median CAP (steatosis) in females	Body mass index	4.66E+00	1.52E−01	1.61E−22	100.595%
		Estrone	1.36E−02	1.76E−02	4.44E−01	NA		Estrone	−7.91E−03	3.94E−03	5.48E−02	NA
5	Median CAP (steatosis) in males	Body mass index	6.12E+00	1.90E−01	4.52E−23	100.476%	Median CAP (steatosis) in females	Body mass index	4.62E+00	1.52E−01	1.89E−22	99.813%
		Estradiol	−2.57E−02	2.06E−02	2.23E−01	NA		Estradiol	−9.44E−03	3.09E−03	5.10E−03	NA
6	Median CAP (steatosis) in males	Body mass index	6.09E+00	1.92E−01	6.83E−23	99.979%	Median CAP (steatosis) in females	Body mass index	4.61E+00	1.55E−01	3.45E−22	99.606%
		Progesterone	−2.32E−01	3.79E+00	9.52E−01	NA		Progesterone	−1.82E−01	6.06E−02	5.73E−03	NA
7	Median CAP (steatosis) in males	Body mass index	6.09E+00	1.93E−01	7.61E−23	99.919%	Median CAP (steatosis) in females	Body mass index	4.61E+00	1.58E−01	5.91E−22	99.472%
		Follicle stimulating hormone	−1.34E−01	1.62E−01	4.16E−01	NA		Follicle stimulating hormone	−3.56E−02	3.37E−02	3.00E−01	NA
8	Median CAP (steatosis) in males	Body mass index	6.09E+00	1.93E−01	7.12E−23	99.948%	Median CAP (steatosis) in females	Body mass index	4.62E+00	1.55E−01	3.68E−22	99.695%
		Luteinizing hormone	−1.56E−01	2.28E−01	4.99E−01	NA		Luteinizing hormone	−5.33E−02	5.19E−02	3.13E−01	NA
9	Median CAP (steatosis) in males	Body mass index	5.67E+00	1.78E−01	5.62E−23	93.049%	Median CAP (steatosis) in females	Body mass index	4.25E+00	1.64E−01	1.22E−20	91.852%
		Sex hormone binding globulin	−4.94E−01	4.64E−02	3.56E−11	NA		Sex hormone binding globulin	−2.37E−01	2.71E−02	2.26E−09	NA

Results of the weighted multivariable linear regression analysis of BMI, 9 sex hormones, and covariates on median CAP (steatosis) in males (left) and females (right). Only the results for BMI and 9 sex hormones in the multivariable analysis are shown; further details can be found in Table S4 (Supplemental Digital Content, https://links.lww.com/MD/Q408). Ratio of BMI’s effect on median CAP represents the effect of BMI on median CAP, comparing the impact after a djusting for sex hormones to the impact without adjustment.

BMI = body mass index, CAP = controlled attenuated parameter.

## 4. Discussion

We investigated the role of sex hormones in mediating the relationship between BMI and liver steatosis using data from the NHANES 2017 to 2023 cohort and found that BMI was significantly correlated with sex hormone levels, which may play a key mediating role in the link between BMI and hepatic steatosis. We found that 3 hormones acted as mediators between BMI and hepatic steatosis in both men and women: 17α-hydroxyprogesterone, FSH, and SHBG. However, some hormones appeared to have gender-specific effects, such as progesterone, which mediated the relationship between BMI and hepatic steatosis in women but not in men. Additionally, our study found significant gender differences in the effect of BMI on sex hormones. For example, in men, BMI was associated with estrone sulfate, androstenedione, and estradiol, whereas no such associations were found in women. We also observed that BMI was correlated with LH in women, but no such correlation was observed in men. Furthermore, we found that the effect of sex hormones on hepatic steatosis varies between genders. For example, in men, androstenedione and estrone sulfate were associated with hepatic steatosis, whereas no such association was observed in women. In contrast, in women, LH, progesterone, and estradiol were associated with hepatic steatosis, but no such correlation was found in men. Our study highlights the complex and gender-specific role of sex hormones in mediating the relationship between BMI and hepatic steatosis, with distinct hormonal associations observed in men and women. We acknowledge that the inability to rule out the impact of genetic factors and metabolic diseases represents a key limitation, as it hinders a more definitive interpretation of why certain sex hormone-BMI-hepatic steatosis associations are evident in men but absent in women. Future studies addressing this confounding issue would greatly enhance the robustness and clinical translatability of research in this field.

Sex differences also play a significant role in hepatic steatosis, with males being more predisposed to its development.^[19–21]^ Our study findings are consistent with previous research, indicating that the association between BMI and median CAP is considerably stronger in males than in females (beta 6.09 vs 4.63). Sex hormones play a critical role in hepatic energy metabolism by modulating processes such as lipid homeostasis, lipogenesis, fatty acid oxidation, and lipolysis.^[10,22]^ Several studies have identified sex hormones as pivotal contributors to the development of hepatic steatosis.^[23–30]^ A study demonstrated that higher SHBG levels are associated with lower fatty liver index in males and females, while observational findings revealed that 17α-hydroxyprogesterone is inversely related to fatty liver index in males, underscoring the distinct roles of SHBG and sex hormones in hepatic lipid accumulation.^[27]^ A study involving 2912 middle-aged and elderly Chinese individuals found a negative association between SHBG levels and non-alcoholic fatty liver disease.^[28]^ In early menopausal women, estrogen therapy has been shown to effectively prevent the development of hepatic steatosis.^[29]^ In a cross-sectional study of 2835 postmenopausal women and 2899 men, higher estradiol levels were significantly associated with an increased risk of fatty liver.^[30]^ Our study found that in both men and women, 17α-hydroxyprogesterone and SHBG are associated with hepatic steatosis, while estrogens exhibit distinctly different effects; for example, in women, estrone sulfate is not associated with hepatic steatosis, estrone shows a positive association, and estradiol an inverse association.

As the most abundant estrogen, estradiol can decreasing triglyceride accumulation in hepatocytes.^[10,31]^ However, there is a paucity of data regarding the direct effects of estrone and estrone sulfate on hepatic steatosis. Although these hormones, together with estradiol, have been included in studies aiming to elucidate the comprehensive impact of estrogens on hepatic lipid metabolism,^[14,32]^ their individual roles remain poorly defined. The distribution of estrogen receptors in the liver shows distinct sexual dimorphism: females have significantly higher ERα expression in hepatocytes compared to males.^[33,34]^ This sex difference in hepatic ERα expression may help explain why estradiol appears unrelated to steatosis in males but tends to show a protective effect in females, as lower ERα levels in males might limit estradiol’s ability to regulate lipid metabolism, while higher ERα in females could support such protective actions. Estradiol may exert its hepatoprotective effects through pathways such as upregulating AQP7, improving insulin resistance, inhibiting fibrosis, or regulating hepatic fatty acid metabolism,^[34–36]^ though further research is needed to validate these in humans and link them to our observed gender-specific mediation.

Consequently, the specific functions of each estrogen are not yet fully clarified, and thus our research provides a distinct perspective for subsequent studies. Our study extends these findings by specifically examining the mediating role of sex hormones in the relationship between BMI and hepatic steatosis, thereby providing a more nuanced insight into the underlying mechanisms.

While our study offers several strengths – namely, the use of a large, nationally representative NHANES dataset with a diverse participant pool and advanced methods such as vibration-controlled transient elastography for accurate liver fat measurement – the primary innovation lies in our application of mediation analysis to elucidate the role of sex hormones in the relationship between BMI and liver steatosis. Despite these strengths, our study has several limitations. First, the cross-sectional design of the NHANES data restricts our ability to infer causal relationships among BMI, sex hormones, and hepatic steatosis. Additionally, although we controlled for several important covariates, there may still be unmeasured confounders that could influence the observed relationships. Finally, fluctuations in female-specific hormones, such as LH and FSH throughout the menstrual cycle, may compromise the reliability of our findings in this subset of analyses.

## 5. Conclusion

Our study demonstrates that BMI is significantly associated with hepatic steatosis, with sex hormones mediating this relationship in a gender-specific manner. Specifically, 17α-hydroxyprogesterone, FSH, and SHBG emerged as mediators in both sexes, while other hormones exhibited divergent effects between males and females. These findings provide refined insights into the intricate interplay among BMI, sex hormones, and liver fat accumulation. Future longitudinal studies are needed to further validate these associations and clarify the underlying causal mechanisms.

## Acknowledgments

We would like to express our sincere gratitude to the National Health and Nutrition Examination Survey (NHANES) for providing access to their invaluable dataset, which was essential for conducting our research.

## Author contributions

**Conceptualization:** Mingxing Zhao, Caifeng Yang.

**Data curation:** Mingxing Zhao, Caifeng Yang.

**Formal analysis:** Mingxing Zhao, Caifeng Yang.

**Funding acquisition:** Mingxing Zhao, Caifeng Yang.

**Investigation:** Mingxing Zhao, Caifeng Yang.

**Methodology:** Mingxing Zhao, Caifeng Yang.

**Project administration:** Mingxing Zhao, Caifeng Yang.

**Resources:** Mingxing Zhao, Caifeng Yang.

**Software:** Mingxing Zhao, Caifeng Yang.

**Supervision:** Mingxing Zhao, Caifeng Yang.

**Validation:** Mingxing Zhao, Caifeng Yang.

**Visualization:** Mingxing Zhao, Caifeng Yang

**Writing – original draft:** Mingxing Zhao, Caifeng Yang.

**Writing – review & editing:** Mingxing Zhao, Caifeng Yang.

## Supplementary Material


